# Everyday Eating Experiences of Chocolate and Non-Chocolate Snacks Impact Postprandial Anxiety, Energy and Emotional States

**DOI:** 10.3390/nu4060554

**Published:** 2012-06-20

**Authors:** François-Pierre J. Martin, Nicolas Antille, Serge Rezzi, Sunil Kochhar

**Affiliations:** BioAnalytical Science, Nestlé Research Center, Nestec Ltd., PO Box 44, CH-1000 Lausanne 26, Switzerland; Email: Francois-Pierre.Martin@rdls.nestle.com (F.-P.J.M.); Nicolas.antille@rdls.nestle.com (N.A.); Serge.Rezzi@rdls.nestle.com (S.R.)

**Keywords:** anxiety state, anxiety trait, chocolate, energy, emotion

## Abstract

Social and psychological stressors are both a part of daily life and are increasingly recognized as contributors to individual susceptibility to develop diseases and metabolic disorders. The present study investigated how snacks differing in sensory properties and presentation can influence ratings of affect in consumers with different levels of dispositional anxiety. This study examines the relationships between a pre-disposition to anxiety and food using a repeated exposures design with three interspersed test days over a period of two weeks. The study was conducted on ninety free-living male (*n* = 28) and female (*n* = 62) Dutch participants aged between 18 and 35 years old, with a BMI between 18 and 25 kg/m^2^ and different anxiety trait levels assessed using State-Trait Anxiety Inventory tests. The study was randomized by age, gender, anxiety trait score, and followed a parallel open design. Three test products: dark chocolate, a milk chocolate snack and crackers with cheese spread (control), which differed in composition, sensory properties and presentation, were evaluated. Changes in self-reported anxiety, emotion, and energetic states were assessed as a function of eating the snacks just after consumption and up to one hour. The repeated exposure design over a period of two weeks enabled the investigations of potential cumulative effects of regular consumption of the food products. The milk chocolate snack resulted in the decrease of anxiety in high anxiety trait subjects, whereas dark chocolate and cheese and crackers respectively improved the anxiety level and the energetic state of low anxiety trait participants. The mood effects were not altered with repeated exposure, and the magnitude of changes was similar on each test day, which illustrates the repeatability of the effects of the food on subjective measures of postprandial wellness.

## 1. Introduction

Social and psychological stressors are both a part of daily life and are increasingly recognized to impact individual traits with inferred effects on metabolic health, emotions and moods, and stress may play a role in determining dietary choices [1]. Several studies have provided evidence that stress exposure influences food intake under laboratory settings [2,3,4,5,6]. Food intake is a complex process under the influence of both physiological aspects (e.g., hormonal regulation of hunger and satiety) and subjective aspects [7]. The later encapsulates senses (e.g., taste, smell, palatability, or texture), cognitive perception (e.g., preferences, aversions) and also the postprandial feeling of wellness perceived by the consumer after the consumption of the product [8]. Moreover, consumers have preconceived ideas about product features that will influence their perceived satiety level, which may subsequently affect their food preference [9]. For instance, products perceived as fat, high in proteins, with a savory taste are expected to have a higher level of satiety compared to sweet products. Previous reports described that stress generally produces differential effects on eating depending on the type of subject. For instance, restrained and emotional eaters tend to overeat in response to stress, whereas the intake of unrestrained and non-emotional eaters remains constant or may decrease [3]. Moreover, stress is often associated with increased consumption of high fat and highly palatable foods [4,10]. Various studies have demonstrated that individuals in a negative mood state may eat in an effort to provide comfort or distraction from negative emotions [6,11,12]. For instance, the intake of chocolate was examined as a means to improve mood [13,14]. While cocoa and chocolate products are perceived as being highly palatable and in some cases hedonistic, they may have also beneficial effects on human metabolism. The high content of flavonoids (catechins and procyanidins) in some cocoa-based products are associated with benefits to cardiovascular health by maintaining low blood pressure, improving endothelial function, and by reducing markers of thrombosis, oxidation and inflammation [15,16,17,18,19]. In addition, theobromine, an alkaloid analogous to caffeine but primarily found in cocoa, is known to reduce blood pressure, and other cocoa alkaloids may potentially influence brain and neural metabolism [14,19,20].

In order to investigate if chocolate-based snacks could have an impact on postprandial wellness, we conducted a clinical trial on healthy Dutch adults using three different snacks and measured postprandial state anxiety, energetic and emotional states. First, we sought to capture if pre-disposition to stress was a determinant of the measured postprandial effects on anxiety, energy and emotional states. We also assessed if regular consumption of a product over a period of two weeks would lead to specific cumulative effects during the postprandial experience (e.g., a short-lived or long-term beneficial improvement of the anxiety perception). Since there is evidence that certain types of foods, particularly carbohydrates, may influence ratings of affects [21,22], three different snack foods were evaluated to cover a larger area of the snack food segment, including a popular milk chocolate snack, a popular and typical savory snack in the study population (cream cracker with cheese spread), and a dark chocolate snack. 

## 2. Experimental Section

### 2.1. Design of Study

The study was designed as a randomized, parallel, open study (Table 1–Table 3). Participants were randomized by age, gender, and self-reported anxiety level. Two between-subject factors were considered; the first was the product consumed during the study, the second factor was dispositional stress (anxiety trait, Table 1). Participants were divided into either high or low anxiety sub-group according to an initial evaluation of their dispositional anxiety as assessed by scoring on the anxiety trait scale of the State-Trait Anxiety Inventory (STAI) test [23]. The STAI enables measuring anxiety in adults which clearly differentiates between the temporary condition of “state anxiety” and the more general and long-standing quality of “anxiety trait”. The essential qualities evaluated by the state-anxiety scale are feelings of apprehension, tension, nervousness, and worry. The STAI test provides an indication of anxiety, depression and neurosis. Respondents indicated on a 4-point scale to what degree they were experiencing a particular state related to anxiety. STAI was measured at the beginning of the study and during the nutritional intervention. STAI scores from 70 to 78, and from 42 to 64, were used to describe low and high anxiety trait participants, respectively. Scores on the questionnaire were used for randomization in order to get an even distribution over the treatment groups. Participants with high or low anxiety levels were randomly assigned to a food condition (milk chocolate snack (*n* = 16 high anxiety trait, 14 low anxiety trait), dark chocolate (*n* = 13 high anxiety trait, 17 low anxiety trait) or cheese and crackers (*n* = 15 high anxiety trait, 15 low anxiety trait), Table 1 and Table 2). Participants were informed of their randomized conditions on the first day of the study (day 01).

**Table 1:** Outline of the study design.

	Duration of consumption	Food condition	Chocolate snack ( *n* = 30)		Dark chocolate ( *n* = 30)		Cheese and Cracker ( *n* = 30)	
		Dispositional stress	HA (* *n* = 16)	LA (* *n* = 14)	HA (* *n* = 13)	LA (* *n* = 17)	HA (* *n* = 15)	LA (* *n* = 15)
Test day 1	Day 01		One-off consumption as mid-morning snack. Evaluation of anxiety, energetic and emotional states before (T0) and after (T10 and T60) intake					
Diary period	Days 02 to 07		Consumption twice daily mid-morning (10:30 a.m.) and mid-afternoon (3:30 p.m.)					
Test day 2	Day 08		One-off consumption as mid-morning snack. Evaluation of anxiety, energetic and emotional states before (T0) and after (T10 and T60) intake					
Diary period	Days 09 to 14		Consumption twice daily mid-morning (10:30 a.m.) and mid-afternoon (3:30 p.m.)					
Test day 3	Day 15		One-off consumption as mid-morning snack. Evaluation of anxiety, energetic and emotional states before (T0) and after (T10 and T60) intake					

* Number of participants per sub-group after randomization by food condition; Run in period: 8 days; HA, high anxiety trait; LA, low anxiety trait.

**Table 2:** Demographic data.

	Low anxiety trait		High anxiety trait		*p*-value
	*N* (M/F)	Mean ± SD	*N* (M/F)	Mean ± SD	
**Food condition 1: Dark chocolate**					
Age (Years)	17 (7/10)	22.6 ± 3	13 (4/9)	23.6 ± 4.8	0.506
BMI (kg/m^2^)		22.1 ± 1.5		21.2 ± 1.9	0.180
Score STAI		73.0 ± 2.3		56.3 ± 4.0	6.88 × 10^−11^
**Food condition 2: Milk chocolate wafer snack**					
Age (Years)	14 (5/9)	22.1 ± 3.3	16 (3/13)	23.9 ± 4.2	0.187
BMI (kg/m^2^)		22.1 ± 1.6		21.9 ± 1.9	0.717
Score STAI		73.6 ± 2.6		58.1 ± 4.8	6.04 × 10^−11^
**Food condition 3: Cheese and cracker**					
Age (Years)	15 (5/10)	21.8 ± 3.2	15 (4/11)	22.8 ± 3.3	0.406
BMI (kg/m^2^)		21.6 ± 1.7		21.3 ± 1.8	0.623
Score STAI		73.2 ± 1.3		59.2 ± 5.2	2.67 × 10^−8^

**Table 3:** Composition of the study products.

Composition	Chocolate snack		Dark chocolate		Cream cracker with cheese spread	
	1.5 bar (3 fingers, 25 g)	100 g	per 20 g	100 g	2 crackers + 15 g cheese spread	100 g
Energy (kcal)	127.5	509	114	567	111	438
Protein (g)	1.7	6.7	1.8	9	4.2	10.2
Carbohydrates (g)	15.8	62.6	5.4	27	10.9	66.7
Fats (g)	6.5	25.6	9.4	47	4.4	14.5

The intervention phase of the study (Table 1) was preceded by an eight-day run-in period during which participants refrained from consuming chocolate or products containing chocolate. Compliance to abstain from chocolate and chocolate-containing products during run-in was monitored upon inquiry on day 01 of the study. This allowed for the greatest possible effects to be found for the consumption of the two chocolate products on the first test day, and thus for determining a baseline for their effects. During a period of two consecutive weeks, participants were asked to consume the test products on a twice-daily basis at home and/or at work. Subjects were asked to report deviations from consumption of the study substances and from dietary restrictions. During this period, subjects kept a diary with entries recording stressful events that occurred during the study and responses to a shortened version of the emotion questionnaire. From the daily records, the number of stressful events was calculated per study day per food condition group as well as the intensity of the event (“very much”, “considerable”, “moderately”, “hardly”). In addition, the reported emotions were scored per study day and per treatment group.

The study consisted of two main data collection periods: single intake on three test days (days 01, 08, 15) and intake for two consecutive periods of one week (days 02 to 07 and days 09 to 14). Each test day was intended to assess the acute effects of the study substances. As the study progressed, the subsequent test days revealed the interaction between the acute effects of the study substances and longer term consumption. Consumption of the study substances during the periods in between the test days were included to study the cumulative effects of study substance consumption over time on the dimensions of interest. The time of consumption of the test foods was standardized for all the participants. On days 01, 08, and 15, test foods were given as a mid-morning snack at the study centre at about 10.00 h with a glass of water. The study substances had to be consumed within 5 min. From day 02 to day 07, and from day 09 to day 14, test foods were consumed at home or at work daily as a mid-morning (around 10:30 a.m.) and a mid-afternoon (around 3:30 p.m.) snack. 

### 2.2. Clinical Trial and Participants

This study was conducted in accordance with the ethical principles of Good Clinical Practice and the Declaration of Helsinki. The protocol was approved by the Medical Ethics Committee METOPP (Tilburg, The Netherlands). From 192 subjects that initially expressed interest in the study, 90 (62 females, 28 males) were enrolled into the study, and received an oral briefing about the aim, the procedures, the constraints, the insurance cover and the financial compensation of the study. Subjects were selected based on medical history, age (from 18 to 35 years), body mass index (BMI, from 18 to 25 kg/m^2^), blood pressure, heart rate, and blood clinical (triglycerides, total-cholesterol, high density- and low density-cholesterol, fasting glucose). Habitual preference for chocolate-based foods was assessed using a dedicated questionnaire (data not shown). The exclusion criteria included psychiatric and gastro-intestinal disorders, smoking, use of medication that may influence appetite and/or sensory functioning, reported slimming or medically prescribed diet. Moreover, to avoid any confounding effects related to hunger, subjects were required to start fasting from 10:00 p.m. the evening prior to the test days. To ensure that no eating disorders have occurred during the study, body weight of the subjects was measured every test day before breakfast. The study also aimed to investigate the metabolic signatures associated with anxiety and response to a dietary intervention, for which the results have been published previously [24]. 

### 2.3. Products

The tested products were milk chocolate covered wafer fingers (Food 1, KITKAT mini^®^, Nestlé Deutschland GmbH, 60523 Frankfurt am Main, Germany), dark chocolate (Food 2, Cailler Noir Intense^®^, 74% cocoa solids, Nestlé Suisse SA, Switzerland), and crackers (Food 3, Jacob’s Cream crackers^®^, Jacob’s Bakery Ltd., UK) with cheese spread. The milk chocolate snack was a KITKAT wafer bar selected for its worldwide popularity. The dark chocolate offers an interesting alternative for sensory experience due to its sweetness, bitterness and its high content of cocoa extracts, which also offers a different palatability. The cheese and cracker snack was chosen for its savory features as opposed to the two other sweet products, as due to its position as a typical snack product in the Dutch population. The portion of each product was adjusted to provide a similar amount of energy (Table 3). The tested products were given to the participants in their commercial form including original packaging and reported composition.

### 2.4. Procedures and Measures

On each test day, anxiety, energy and emotional states were measured using subjective reports to questionnaires before (T0, 0 min) and after (T10, 10 min, and T60, 60 min) the intake of the tested products. The anxiety state level was assessed using the STAI [23]. Respondents indicated on a 4-point scale to what degree they were experiencing a particular state related to stress and anxiety. The perceived energy levels were measured using the scale of experienced load [25] to measure the degree to which participants felt sleepy, alert, and interested. Participants responded to items in which the anchors were represented by opposing energy states. Respondents indicated on a 4-point scale where their present energy state lied relative to the two anchors. Emotional state was measured using a classification validated by Richins [26] on a 4-point scale from extremely negative to extremely positive emotions. On each test day, the participants underwent additional cognitive tests administered between T30 and T60 after consuming the snack. The results on cognitive performance are not reported in the present work, since no significant effects and trends were observed.

### 2.5. Analysis

Structural equation modeling (SEM), a statistical analysis for testing and estimating causal relationships using a combination of statistical data and qualitative causal assumptions, was applied for data analysis. The scale reliability was assessed for each of the three scales (anxiety, energy and emotional states) according to a previously published method [27]. Unreliable items or items with little discriminative value were removed from the scales and not used for further analyses using a three step approach. First, the variance of the responses was examined for response biases (e.g., ceiling effects), by subjectively viewing the distributions and removing items in which only one or two response categories were used or in which the distribution was particularly skewed. Second, factor analysis (FA) of the remaining items was applied separately for the three scales (anxiety, energy and emotional states). Each analysis resulted in one usable and interpretable factor summarizing a sub-set of items from the total scale. Third, the coefficient of reliability Cronbach’s α was generated for each sub-scale. The state anxiety scale had an α < 0.85, the energy scale had an α < 0.82 and the emotion scale had an α < 0.81. The high values of Cronbach’s α indicate that the new scales are highly reliable. Therefore, further analysis was only performed only on the items remaining after FA. 

These items were averaged separately for anxiety, energy and emotional states, resulting in three scores for each participant, which were used as the dependent variables for subsequent analysis by ANOVA (SAS V8/V9 Software package, SAS Institute, Cary, NC). The design tested was: Food condition × Anxiety trait × Time of measurement × Test day, with repeated measures on the last two factors. If the analysis of variance indicated a food condition effect (*p* < 0.05), comparisons between the means of the parameters were performed using a paired *t*-test. The null hypothesis was formulated separately for each variable, and food condition effects were analyzed for each variable. The analysis was performed to assess the effects in relation to differences between food conditions, anxiety trait groups and food condition/anxiety trait interaction. Within and between day effects were assessed by ANOVA for analyzing the differences between the baseline measurements (T0) of the different test days, and the differences between baseline measurements (before consumption of the study products) and the measurements taken after consumption of the study snacks on the different test days.

## 3. Results

### 3.1. Anxiety Trait and Groups

The population enrolled in the study included 28 males and 62 females distributed across two anxiety trait levels; high anxiety (33 females and 11 males) and low anxiety (29 females and 17 males). Chi-square tests showed that the observed distribution did not differ significantly from the ideal distribution across the two anxiety states (χ^2^(1) = 1.50, *p* = 0.210). There was no significant difference in gender balance between the two groups. *t*-Tests showed that mean anxiety trait score differed significantly between the two anxiety trait groups (*p* < 0.001): the high anxiety trait participants were significantly more anxious than the low anxiety trait participants. Participants were randomly distributed across the three food conditions (Table 2). Only differences between anxiety trait were observed for each food conditions, age and BMI being not significantly different. The body weight of the subjects was measured every test day before breakfast, and no significant differences were found in body weight during the study and between treatment groups (data not shown). Chi-square analysis of the stressful events data (number of events, intensity of these events and emotions) did not indicate any significant differences during the study.

### 3.2. Short-Term Effects of Snack Intake on Anxiety State

**Figure 1:**
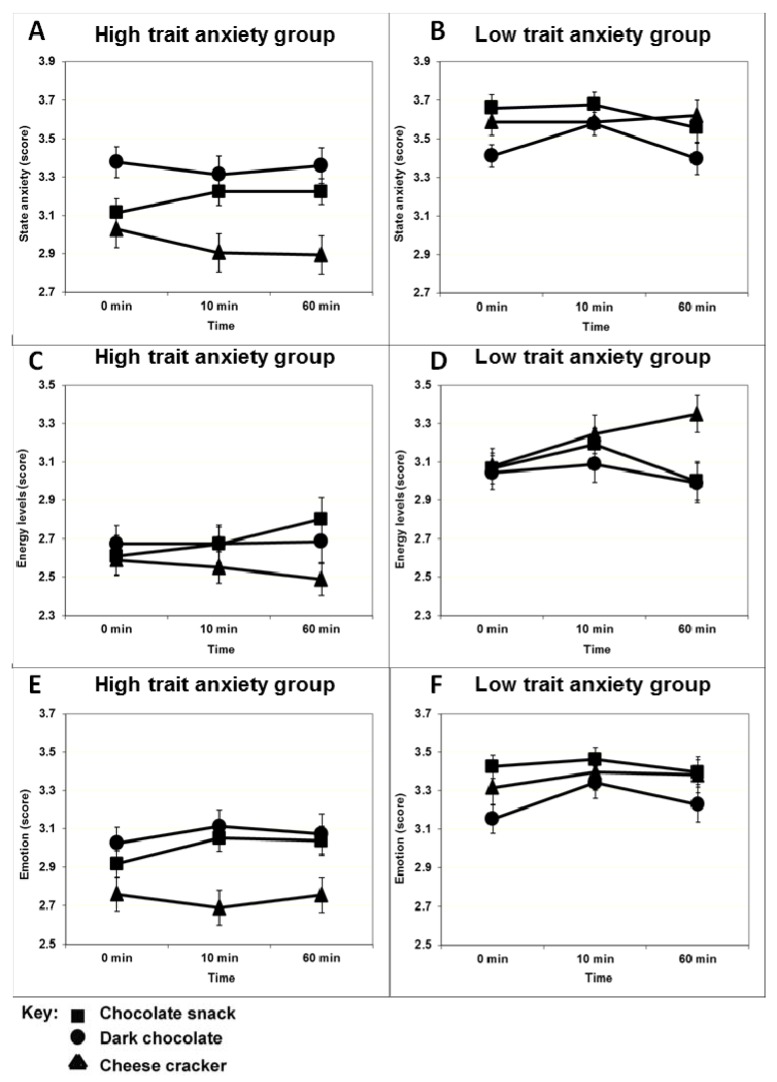
Mean (±SEM) state anxiety score (**A**,**B**), perceived energy levels score (**C**,**D**), and perceived emotion score (**E**,**F**), per food condition (square, milk chocolate snack; dot, dark chocolate snack; triangle, cheese and cracker snack) reported by participants in the high (**A**,**C**,**E**) and low (**B**,**D**,**F**) anxiety trait groups. The mean of the scores was generated from the data collected from the three test days (since no differences were observed over the days). A higher reported score is associated with lower experienced anxiety, a higher energy state and better emotional state.

Multivariate data analysis using SEM and ANOVA found a three-way interaction for anxiety state between anxiety trait, food condition and time (*F*(4,504) = 4.16, *p* = 0.002). Detailed analysis showed that the effect is the result of changes in the levels of state anxiety for the high anxiety trait participants over time. The mean (±standard error of mean, SEM) for the state anxiety score per food condition at different time points for high and low anxiety trait participants are shown in Figure 1. High anxiety trait participants receiving the milk chocolate snack on the test days experienced less anxiety 10 min after consumption compared to baseline, as indicated by higher anxiety state scores (Figure 1). This effect is maintained until one hour after consumption (*t* = 0/*t* = 10: *p* = 0.048; *t* = 0/*t* = 60: *p* = 0.048; *t* = 10/*t* = 60: *p* = 1.000). The participants receiving the cheese and cracker snack experienced more anxiety after consumption compared to the baseline, as indicated by lower scores. This effect is maintained for at least one hour after consumption (*t* = 0/*t* = 10: *p* = 0.032; *t* = 0/*t* = 60: *p* = 0.020; *t* = 10/*t* = 60: *p* = 0.850). No significant effects were observed with dark chocolate in high anxiety trait participants or with any products in the low anxiety trait group (*p* > 0.05). 

### 3.3. Short-Term Effects of Snack Intake on Perceived Energy Levels

Multivariate data analysis using SEM and ANOVA indicated a similar three-way interaction between perceived energy state, food condition and time (*F*(4,504) = 3.30, *p* = 0.011). Detailed analysis showed that the effect is the result of changes in the levels of perceived energy for the high anxiety trait participants as well as for the low anxiety trait participants over time. The mean (±SEM) for the perceived energy score per food condition at different time points for high and low anxiety trait participants are shown in Figure 1. Statistical analysis found that only the intake of the milk chocolate snack resulted in an improvement of energy states in high anxiety trait individuals, 60 min after consumption compared to baseline (*t* = 0/*t* = 10: *p* = 0.490; *t* = 0/*t* = 60: *p* = 0.033; *t* = 10/*t* = 60: *p* = 0.150). Low anxiety trait participants receiving the milk chocolate snack felt that they had lower energy levels 60 min after consumption compared to 10 min after consumption (*t* = 0/*t* = 10: *p* = 0.190; *t* = 0/*t* = 60: *p* = 0.500; *t* = 10/*t* = 60: *p* = 0.047), while those consuming the cheese and crackers had the perception of higher energy levels 60 min after consumption compared to baseline (*t* = 0/*t* = 10: *p* = 0.074; *t* = 0/*t* = 60: *p* = 0.003; *t* = 10/*t* = 60: *p* = 0.260). No significant results were observed for an effect of dark chocolate consumption on perceived energy levels. 

### 3.4. Short-Term Effects of Snack Intake on Perceived Emotional State

There was no short-term effect of the snacks on perceived emotional state (*F*(4,504) = 1.71, *p* = 0.147). The mean (±SEM) for the emotion score per food condition at different time points for high and low anxiety trait participants are shown in Figure 1. 

### 3.5. Cumulative Effect over Time

Mood effects were not altered with repeated exposure, and the magnitude of changes was similar on each test day, illustrating the repeatability of the snack intervention effects on the measures of postprandial wellness (data not shown).

## 4. Discussion

The present investigation is a first attempt to assess the impact of the consumption of different snack products on postprandial anxiety, emotional and energetic states in relation to predisposition to anxiety trait in healthy subjects. This study brings evidence that a predisposition to high or low anxiety traits has an impact on food experience, even in healthy volunteers. These effects were observed between 10 and 60 min after consumption of the snack intervention, with main effects on anxiety.

In the present study, all the food products were presented in their commercial form. The three products differed in term of extrinsic (e.g., brand name, packaging) and intrinsic cues (e.g., sensory characteristics). The questionnaire given to determine preference for chocolate (data not shown) indicated that all subjects showed a high liking score for chocolate-based snacks, whilst cheese and cracker snack was chosen as it is a typical Dutch snack. Because of the different visual appearance of the snacks, consumers’ expectations may vary and therefore either enhance or degrade the perception of the product before its consumption [28]. Recent studies described how exposure to images of chocolate could affect cravings and negative effects such as guilt, anxiety and depression [29,30]. Consumers are also more prone to like a snack due to product appearance before and during consumption [31]. Therefore, the observed results are the consequences of experiencing the product as a whole, including the wrapping, product visional appearance, tactile ad textural factors, taste as well as any physiological effects of the snack components. Moreover, since the study was not blinded, individual perception and preference for the tested product might also be a limitation of the study design, which may explain group specific effects.

The different responses observed between high and low anxiety trait subjects for a given product may be at least partially ascribed to pre-dispositional mood states weight, which differently affect product acceptance. Emotions differing intensity are reported to exert different influences on eating behavior [12,14]. However, the influence of negative emotions on food intake is complex [32], and may decrease or increase food intake depending on individuals [33]. Scheider *et al.* previously investigated how anxiety trait may impact lean and obese individuals’ predisposition to emotional eating [33], and showed a positive association with food intake only in obese subjects. Eating patterns are also known to be influenced by both individual differences in responsiveness to food cues and the emotional state [34]. For instance, Loxton *et al.* showed that the urge to eat was decreased following negative mood induction, but subsequently increased following exposure to food cues [34]. Even if food intake was not measured in the current study, our data suggest that the consumption of the three foods is influenced by pre-dispositional anxiety levels. The results observed with the high anxiety trait population showed that only the milk chocolate snack resulted in positive effects on their anxiety and energetic states only immediately after consumption, whereas the cheese and cracker had a negative impact on anxiety state. With the current experimental design, it is difficult to understand the negative effect of the cheese and cracker snack. All the groups were matched for BMI, and participants were required to be fasted for the same period of time before the test. Pre-dispositional anxiety may have selectively impacted hunger, satiety and food craving, which subsequently influenced the whole eating experience. Such parameters should be considered in follow-up studies to better assess if mood modulation results in the negative reinforcement of food on hunger state. Low anxiety trait subjects are more likely to feel positive emotions, and their response to the food consumption may correspond to a different mood-food acceptance relationship, when compared to high anxiety trait subjects. Interestingly, low anxiety trait individuals tended to experience a decrease in perceived anxiety state immediately after dark chocolate consumption (*p* = 0.140, data not shown), even if changes were not statistically significant. However, the observation of these short lived effects (up to 10 min after intake) is corroborated by other studies suggesting that any improvement of emotions due to food intake occurred during consumption and/or immediately after intake, with negative emotions returning shortly afterwards [14,35]. 

In addition to perceived anxiety and emotions, the sheer complexity of the relationships between pre-dispositional mood, snack product, and postprandial response also brings into play the oro-sensory properties of the product before and during consumption (e.g., taste, smell, sweetness, palatability, and texture) [11,36,37] as well as expectation of satiety [38]. For instance, certain types of foods, particularly sweet foods, influence ratings of affects [21,22,39], whilst product palatability is strongly related to the improvement of negative emotional states [12]. The relationship between emotions and the composition of consumed food is complex, and foods differing in fat and carbohydrate content may induce a large variety of effects on mood [30,40]. Since the snack products used in this study had different sensory properties (sweet *vs*. savory), palatability, macronutrient concentrations, these parameters may have a strong incidence on the group-product specific responses. This also may be the case in this study as an explanation for the differential effects of the snacks on high anxiety trait subjects, even if overall energy content was matched.

Most of the energy in the snacks is provided by fat in dark chocolate, and from carbohydrates in the milk chocolate snack and the cheese and crackers. Tasting foods containing sugars evokes positive affective responses [41], and can have some immediate effects on alertness and make individuals feel more relaxed [42]. Moreover, participants experiencing negative emotions can feel satisfied, happy and relaxed immediately after the consumption of foods containing carbohydrates [43]. The cheese and cracker snack, which has a higher carbohydrate/fat ratio compared to the two chocolate-based snacks, had a negative impact on anxiety state. Therefore, our observations may imply that for alleviating anxiety perception, high anxiety trait subjects are more responsive to the consumption of the milk chocolate snack, due to its oro-sensory properties resulting, at least partially, from its macronutrient composition. 

Perhaps surprisingly, the mood effects were not altered with repeated exposure to the snacks, and the magnitude of changes was similar on each test day, which illustrates the repeatability of the effects of the snack intervention on postprandial wellness without any overall change to mood outside of snacking occasions (data not shown). We have previously reported how metabonomics was employed to measure the subjects’ metabolic responses attributed to daily dark chocolate consumption, using urine and plasma samples collected during this clinical trial [24]. The daily intake of dark chocolate resulted in subtle and cumulative metabolic effects on cortisol and catecholamines which was strongly dependent on the predisposition to stress of the individuals. This was observed via a decrease of the urinary levels of catecholamines (adrenaline, noradrenaline, normetanephrine), corticosterone, and the stress hormone cortisol in high anxiety trait subjects. In addition, within two weeks of consumption, dark chocolate had a positive impact on stress-associated metabolism restoring high stress metabolic features (energy, microbial activity and hormonal metabolism) towards the levels found in less stressed subjects, though no variation in self-reported anxiety was reported over the time of study. These additional metabonomics-based observations illustrate how snack foods may determine a large variety of effects on human metabolism, including immediate and short-lived effects as well as long-term biochemical modulation of the stress and energy metabolism. Long-term studies are needed to see if changes to stress hormone levels and metabolic profiles can ultimately lead to variations in perceived anxiety. Moreover, the current experimental design has several limitations, which obscure data interpretation due to differences in crispy-creaminess, packaging, and calories for instance. In addition, since the study aimed at investigating the interactions between food product and pre-dispositional anxiety in free-living subjects, it was not feasible to correct for the total energy intake of the subjects, menstrual cycle in the women or for eating behavior, which are additional confounding factors. However, the study provided novel insights into the consumer perception of different snack products on postprandial wellness, which can be followed-up in future studies.

## 5. Conclusions

The observations from this study demonstrate how snacks differing in sensory properties and presentation can influence the postprandial experience of consumers with low or high anxiety trait over a period of two weeks. The milk chocolate snack resulted in the decrease of anxiety in high anxiety trait subjects, whereas low anxiety trait participants could perceive benefits on energetic state with cheese and crackers. The mood effects were not altered with repeated exposure, and the magnitude of changes was similar on each test day, which illustrates the repeatability of the effects of the food on the postprandial wellness. Additional investigations are required to assess the respective contribution of other factors, such as the satiety, food craving, hunger, separation of individual snack components, pre-dispositional emotional and energetic states, to understand how the milk chocolate snack may improve emotional experience among individuals with high anxiety trait. 
